# Targeted Decorin Gene Therapy Delivered with Adeno-Associated Virus Effectively Retards Corneal Neovascularization *In Vivo*

**DOI:** 10.1371/journal.pone.0026432

**Published:** 2011-10-19

**Authors:** Rajiv R. Mohan, Jonathan C. K. Tovey, Ajay Sharma, Gregory S. Schultz, John W. Cowden, Ashish Tandon

**Affiliations:** 1 Harry S. Truman Memorial Veterans' Hospital, Columbia, Missouri, United States of America; 2 Mason Eye Institute, University of Missouri-Columbia, Columbia, Missouri, United States of America; 3 College of Veterinary Medicine, University of Missouri-Columbia, Columbia, Missouri, United States of America; 4 Department of Ophthalmology and Obstetrics and Gynecology, University of Florida, Gainesville, Florida, United States of America; University of Chicago, United States of America

## Abstract

Decorin, small leucine-rich proteoglycan, has been shown to modulate angiogenesis in nonocular tissues. This study tested a hypothesis that tissue-selective targeted decorin gene therapy delivered to the rabbit stroma with adeno-associated virus serotype 5 (AAV5) impedes corneal neovascularization (CNV) *in vivo* without significant side effects. An established rabbit CNV model was used. Targeted decorin gene therapy in the rabbit stroma was delivered with a single topical AAV5 titer (100 µl; 5×10^12^ vg/ml) application onto the stroma for two minutes after removing corneal epithelium. The levels of CNV were examined with stereomicroscopy, H&E staining, lectin, collagen type IV, CD31 immunocytochemistry and CD31 immunoblotting. Real-time PCR quantified mRNA expression of pro- and anti-angiogenic genes. Corneal health in live animals was monitored with clinical, slit-lamp and optical coherence tomography biomicroscopic examinations. Selective decorin delivery into stroma showed significant 52% (p<0.05), 66% (p<0.001), and 63% (p<0.01) reduction at early (day 5), mid (day 10), and late (day 14) stages of CNV in decorin-delivered rabbit corneas compared to control (no decorin delivered) corneas in morphometric analysis. The H&E staining, lectin, collagen type IV, CD31 immunostaining (57–65, p<0.5), and CD31 immunoblotting (62–67%, p<0.05) supported morphometric findings. Quantitative PCR studies demonstrated decorin gene therapy down-regulated expression of VEGF, MCP1 and angiopoietin (pro-angiogenic) and up-regulated PEDF (anti-angiogenic) genes. The clinical, biomicroscopy and transmission electron microscopy studies revealed that AAV5–mediated decorin gene therapy is safe for the cornea. Tissue-targeted AAV5-mediated decorin gene therapy decreases CNV with no major side effects, and could potentially be used for treating patients.

## Introduction

Corneal neovascularization (CNV) is a sight-threatening condition resulting from various corneal insults such as infection, trauma, chemical burns, inflammation, limbal insufficiency, allergic eye diseases, etc [1], [2]. Over 4% of the US population is estimated to have CNV, with 1.4 million Americans developing this disorder annually and 12% of these patients suffers with decreased visual acuity [3]. The clinical management of CNV represents a formidable challenge as current pharmacotherapeutic and surgical options are associated with serious complications, are not always effective, and may fail altogether necessitating corneal transplant. Currently, corticosteroids form the cornerstone for treating CNV but are at times ineffective and may lead to adverse side effects including cataracts, glaucoma, and infection [4]. More recently, an anti-vascular endothelium derived growth factor (VEGF) antibody has been tested in patients to treat CNV [5]. In addition, a few other anti-angiogenic proteins have been evaluated to treat CNV [6], [7]. Nonetheless, all current therapies to treat CNV are inefficient, provide only short-term relief, cause serious side effects and are often ineffective. Furthermore, the therapeutic efficacy of topical ophthalmic drugs is greatly compromised due to the drug's poor permeability to the cornea, inability to permeate the corneal epithelial barrier, and rapid turnover due to tear fluid and nasolacrimal drainage [8].

Gene therapy is an attractive approach to treat CNV. The cornea is an ideal organ for gene therapy because of its immune-privileged status, ease of administering gene therapy reagents and visual monitoring. The potential of gene therapy to treat CNV has been demonstrated using genes such as angiostatin, PEDF, Flt-1, and soluble vascular endothelium growth factor receptor [9]–[12]. However, ineffective delivery of therapeutic genes into targeted cells often leads to numerous side effects and sharply limits clinical usefulness of gene therapy in patients. We identified multiple adeno-associated virus (AAV) serotypes efficient and safe for delivering foreign genes into rodent, rabbit, equine, and human cornea *in vitro* and *in vivo* [13]–[18]. Other investigators also found AAV effective for delivering genes in the cornea [10], [19], [20]. Recently, we demonstrated tissue-targeted gene delivery in the stroma of the normal and damaged (hazy or neovascularized) rabbit cornea *in vivo* using a combination of AAV5 and custom vector-delivery techniques [Mohan RR, et al. Invest Ophthalmol Vis Sci. 2005, 46 E-Abstract 2163]. Gene delivery into stroma with this method did not cause any visual short-term side effects in the rabbit eye. It has been our hypothesis that AAV-mediated targeted delivery of anti-angiogenic genes in the corneal stroma effectively decrease CNV without causing significant side effects.

The cornea contains numerous pro-angiogenic and anti-angiogenic factors, and a shift in the balance between these two factors triggers blood vessel growth in the avascular cornea [1], [2]. The precise molecular mechanism causing CNV is still unknown. Vascular endothelial growth factor (VEGF) and basic fibroblast growth factor (FGF2) are known to induce CNV *in vivo* [1], [2]. Sprouting of new blood vessels in the cornea towards an implanted VEGF or FGF2 pellet strongly supports this notion. Decorin, a small leucine-rich proteoglycan, has been shown to be expressed in the cornea and is known to play a vital role in fibrillogenesis and extracellular matrix (ECM) and angiogenesis regulation [21], [22]. Recent research has demonstrated that decorin suppresses endothelial migration as well as the formation of vascular tubes, implicating it's role in angiogenesis [23], [24]. Sulochana et al. showed anti-angiogenic properties of decorin are mediated by the inhibition of endothelial cell migration [25]. Further support for decorin's anti-angiogenic properties comes from an *in vivo* study performed in nude mice that demonstrated decreased angiogenesis in tumor xenografts that over-express decorin [26]. These literary reports prompted us to hypothesize that targeted delivery of decorin into rabbit stroma with recently defined tissue-selective gene transfer technique would effectively inhibit CNV with minimal side effects. This study tested the hypothesis by introducing decorin gene into the rabbit cornea stroma with AAV5 via a custom vector delivery technique after CNV was induced with VEGF using a micro-pocket assay.

## Results

### Validation of Decorin Gene Delivery

**Figure 1:**
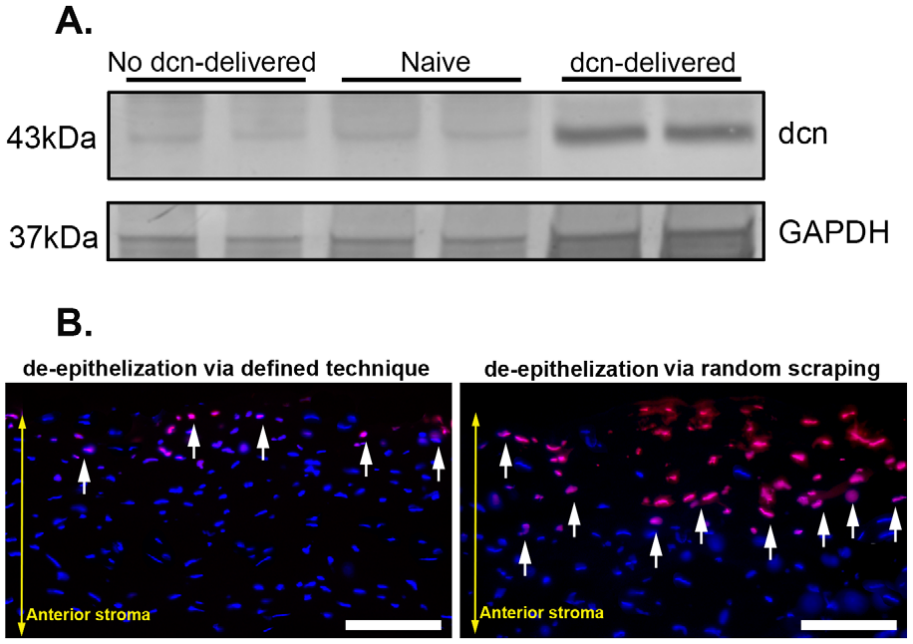
Representative immunoblot (A) and TUNEL assay (B) of rabbit corneas receiving AAV5-decorin or AAV5-gfp. Representative western blotting showing AAV5-controlled decorin delivery in rabbit corneas collected 14 days after VEGF-implantation (A) and TUNEL assay detecting keratocyte apoptosis in rabbit corneas collected 4 hours after epithelial removal by two different ways (B). A strong band in AAV5-dcn treated rabbit corneas of day-14 time point reveals significant decorin delivery (8.7-fold, p<0.05) in the stroma, and a weak decorin band in naive or AAV5-gfp treated corneas indicates low endogenous decorin expression (A). GAPDH was used to confirm equal protein loading in each well and normalization of the data. TUNEL-stained rabbit corneal sections of 4-hour time point shown in Panel B demonstrate minimal keratocyte apoptosis (arrow) in cornea deepithelialized via gentle scrapping with #64 surgical blade by running blade at 45° angle (left panel) compared to high keratocyte death in cornea in which epithelium was removed in random manner with #64 surgical blade (right panel). Scale bar denotes 100 µm. dcn = decorin.

The delivery of decorin gene with a single topical application of AAV5-dcn vector in the rabbit cornea was validated with western blotting. Figure 1A shows the levels of detected decorin in naive, control (no decorin) and decorin-delivered rabbit corneas. The protein lysates prepared from the AAV5-dcn treated corneas showed significant 8.7±0.4 (p<0.05) fold higher levels of decorin confirming substantial delivery of therapeutic gene, decorin, in the rabbit corneas with selected AAV serotype. Contrary to this, very weak bands of decorin detected in the naive and naked-AAV5 vector treated rabbit corneas confirmed the low endogenous expression of decorin protein in the rabbit cornea.

Injury to corneal epithelium has been shown to cause keratocyte apoptosis as well as infiltration of inflammatory, bone marrow derived and other cells in the stroma [27], [28]. TUNEL data shown in Figure 1B demonstrates that defined corneal deepithelialization technique (gentle removal of corneal epithelium by running #64 surgical blade at 45° angle) for gene therapy induces minimal keratocyte death in rabbit corneas collected 4 hours compared to the corneas in which epithelium was removed using #64 blade with no particular angle, pattern or care. As evident from Figure 1B, rabbit corneal sections of defined technique showed <5% TUNEL+ cells (left panel) in the stroma compared to the sections of the rabbit corneas in which epithelium was removed by fast and random scraping (right panel). As expected, naive corneas showed TUNEL+ cells only in epithelium (data not shown).

### Morphometric quantification of CNV in Decorin-delivered and Non-delivered Rabbit Eye

**Figure 2:**
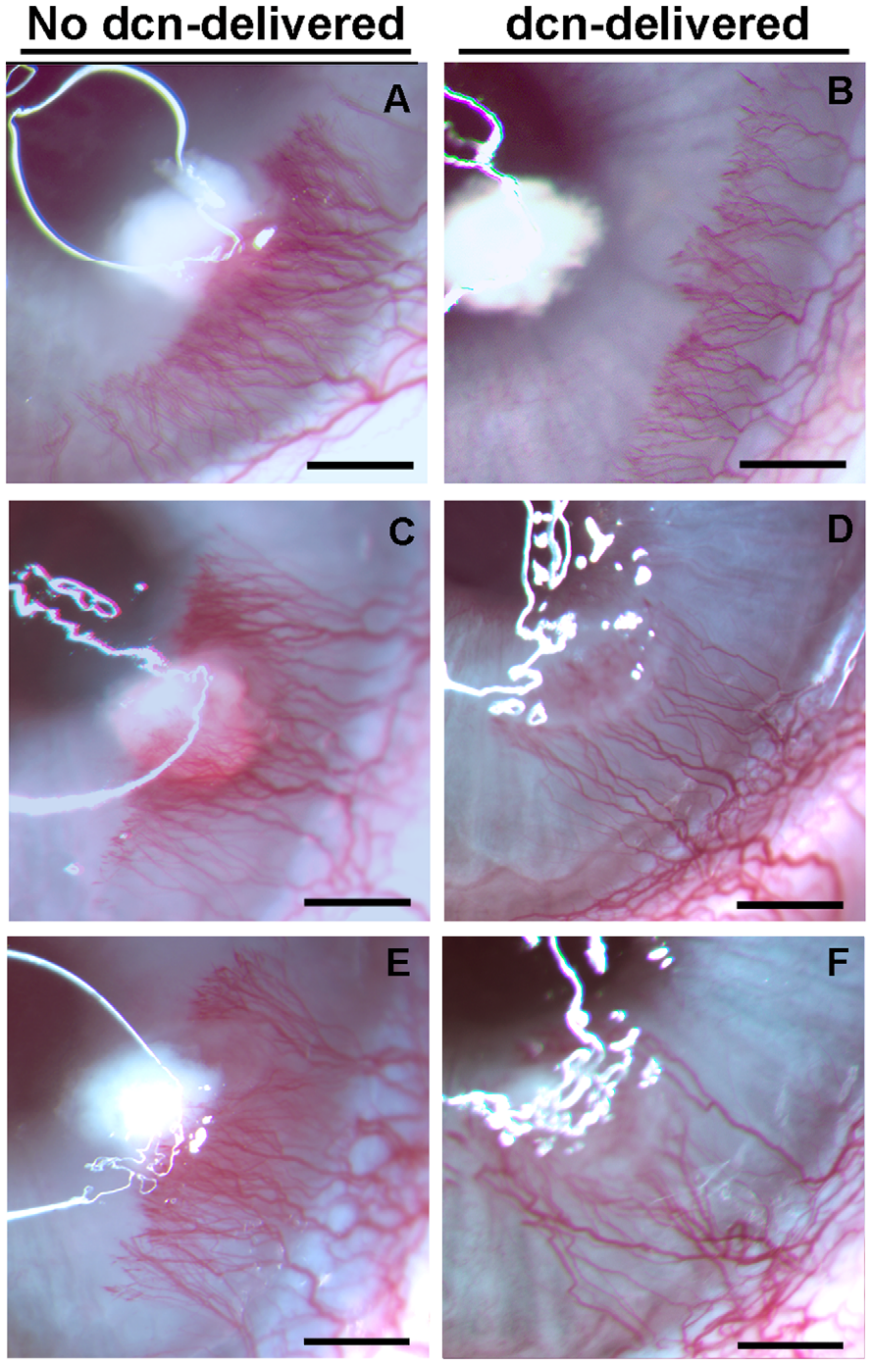
Representative stereomicroscopy images showing VEGF-induced CNV in no decorin-delivered control (A, C and E) and decorin-delivered (B, D and F) rabbit corneas. Rabbit eyes were imaged early (5 day, Panels A, B), mid (10 day, Panels C, D), and late (14 day, Panels E, F) stages after VEGF pellet implantation. The 100 µl AAV5 viral titer (5×10^12^ vg/ml) expressing no gene/gfp or decorin was topically applied onto the cornea after removing corneal epithelium for single application for 2 minutes. A statistically significant inhibition of neovascularization was observed at three tested early, mid and late stages of the CNV. Scale bar denotes 2 mm. dcn = decorin.

**Figure 3:**
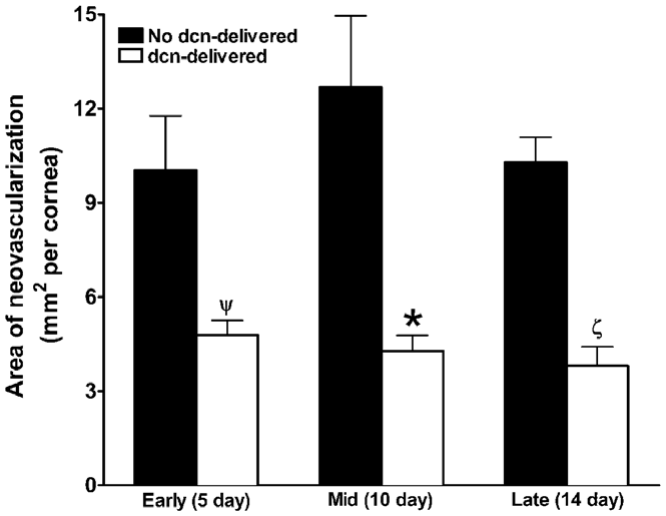
Morphometric quantification of CNV from different time points in rabbit eyes receiving AAV5+/−decorin. Data was collected after day 5 (early), 10 (mid), and 14 (late) of VEGF implantation from the control (no decorin-delivered) and decorin-delivered rabbit corneas. AAV-mediated decorin gene therapy demonstrated statistically significant decrease at the three tested stages of the CNV in rabbit model *in vivo*. ψ = p<0.05, * = p<0.001, ζ = p<0.01 compared to control. dcn = decorin.

Figure 2 shows stereomicroscopic images depicting the area, length and density of VEGF-induced blood vessels in decorin-delivered and no decorin-delivered (control) rabbit corneas observed at 3 different time points. Implanted VEGF produced a strong angiogenic response as evidenced by the presence of blood vessels in avascular regions of the cornea. The peak angiogenic response in control corneas that received AAV5-naked or AAV5-gfp vector was observed on day 10. Decorin-delivered rabbit corneas showed significant decrease in the area, length, thickness and density of the blood vessels at the early- (day 5; Fig 2B), mid- (day 10; Fig 2D) and late- (day 14; Fig 2F) stages of CNV compared to corresponding control (no decorin-delivered) corneas (Fig. 2A, 2C and 2E). To quantify the efficacy of AAV5-mediated decorin gene therapy, area, length and density of blood vessels in the cornea was determined with NIH software Image J. Figure 3 shows mean corneal vasculature area detected in decorin-delivered and control (no decorin-delivered) rabbit corneas at three time points namely 5, 10 and 14 day which represent early, mid and late stages of CNV, respectively. The control corneas (AAV5-naked or AAV5-GFP treated) showed a mean vasculature area of 10 mm^2^ at day 5, 12.7 mm^2^ at day 10, and 10.3 mm^2^ at day 14. Contrary to this, decorin-delivered rabbit corneas showed mean CNV area of 4.8 mm^2^ on day 5, 4.3 mm^2^ on day 10, and 3.8 mm^2^ on day 14. The relative comparison of CNV data between the control and decorin-delivered corneas revealed a statistically significant reduction in corneal neovascularization by 52±5.5% on day 5 (ψ = p<0.05), 66±6.5% on day 10 (* = p<0.001), and 63±6.3% on day 14 (ζ = p<0.01) (Fig. 3).

### Histological comparison of CNV in Decorin-delivered and Non-delivered Corneal Tissues

**Figure 4:**
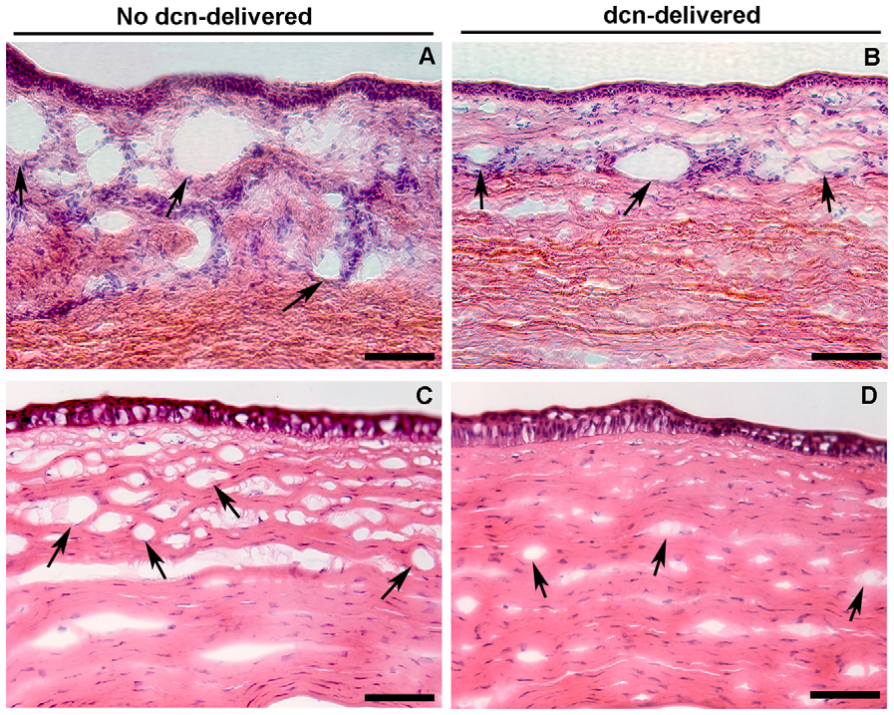
Representative H&E staining images showing anti-angiogenic efficiency of decorin gene therapy in rabbit corneas. The decorin-delivered rabbit corneas (B and D) showed a statistically significant (p<0.01) reduction in vasculature area, density, and blood vessels number, length and diameter compared to control (no decorin-delivered) rabbit corneas (A and C) collected 14 days after VEGF implantation. The images shown in panels A and B are from the corneal sections obtained from the peripheral region of the cornea closer to the limbus whereas the images shown in panels C and D are from the corneal sections proximal to the VEGF pellet. Scale bar denotes 100 µm. dcn = decorin.

Figure 4 shows localization and density of blood vessels in control and decorin-delivered tissue sections of rabbit corneas collected 14 days after VEGF-pellet implantation. Serial sections were prepared. As expected, corneal sections obtained from the peripheral region of the cornea closer to the limbus showed many large-diameter blood vessels (Fig 4A and 4B) whereas corneal sections prepared from the region closer to VEGF pellet showed numerous small-diameter blood vessels (Fig 4C and 4D). The H&E staining performed in the corneal sections of the AAV5-naked and AAV5-dcn vector groups obtained from the same peripheral regions showed markedly less number and reduced-diameter blood vessels in the decorin-delivered rabbit corneas (Fig 4B) compared to the control cornea (Fig. 4A). Similar anti-angiogenic effects of decorin gene therapy were noted in the H&E stained corneal sections prepared from the region closer to the VEGF pellet as decorin-delivered rabbit corneas (Fig 4D) demonstrated significantly (p<0.01) fewer and thin-diameter blood vessels compared to control corneas (Fig. 4C).

**Figure 5:**
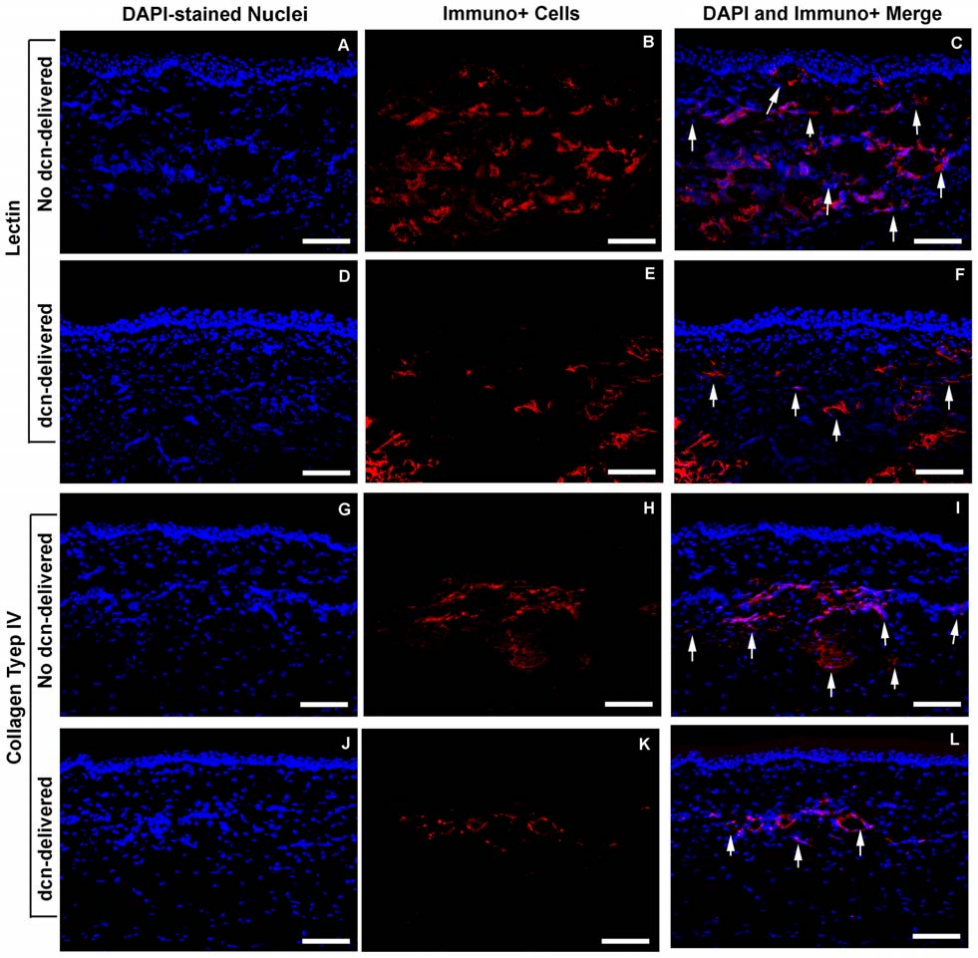
Representative images showing CNV in rabbit corneas treated AAV5+/−decorin. Lectin (A–F) and collagen type IV (G–L) immunostaining was carried out in corneal tissue sections obtained from AAV5-gfp and AAV5-dcn treated rabbit corneas collected 14 days after VEGF implantation. As expected very high lectin (A–C) and collagen type IV (G–I) immunostaining was detected in the corneal sections of AAV5-gfp-treated (no decorin-delivered) corneas due CNV induced by the VEGF implantation. The AAV5-mediated decorin gene therapy significantly reduced (p<0.01) the CNV as evident from the markedly less lectin+ (D–F) and collagen type IV+ (J–L) cells in decorin-delivered rabbit corneal sections. DAPI stained nuclei are shown in blue whereas lectin and collagen type IV+ cells in red. Scale bar denotes 100 µm. dcn = decorin.

The inhibitory effects of decorin gene therapy on CNV were further confirmed by performing immunostaining of angiogenesis markers in the tissue sections of rabbit corneas collected 14 days after VEGF-pellet implantation. Tomato lectin specifically stains blood vessels by binding to the components of basement membrane whereas collagen type IV is a component of basal lamina of blood vessels and CD31 is another endothelial marker. Figure 5 shows the results of lectin (Figs. 5A–F) and collagen type IV (Figs. 5G–L) immunostaining performed in no-decorin (AAV5-gfp-treated) and decorin-delivered (AAV5-dcn-treated) rabbit corneas collected on day-14. Significantly less lectin+ (Fig 5E and 5F; 6,859±393 pixels/200x magnification, p<0.01) and collagen type IV+ (Fig 5K and 5L; 1,983±187 pixels/200x magnification, p<0.01) cells were detected in decorin-delivered rabbit corneas compared to no decorin-delivered control corneas stained for lectin (Fig 5B and 5C; 26,608±713 pixels/200x magnification) or collagen type IV (Fig 5H and 5I; 6,646±259 pixels/200x magnification). The digital quantification of lectin+ and collagen type IV+ cells detected 59–65% (p<0.01) decrease in VEGF-induced corneal vasculature by AAV5-dcn gene transfer in rabbits *in vivo*. Immunostaining using another endothelial marker, CD31, showed similar results (data not shown). These immunohistochemistry investigations suggest that AAV5-mediated decorin gene therapy is highly effective in reducing neovascularization in the rabbit experimental model of CNV.

### Immunoblotting Quantification of CNV in Decorin-delivered and Non-delivered Corneas

**Figure 6:**
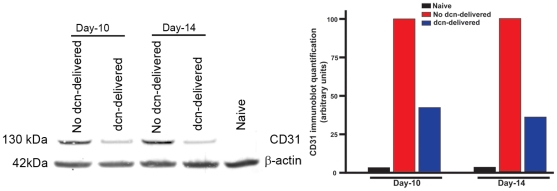
CD31 Western blot for naive, AAV5+/−decorin-delivered rabbit corneal tissues collected 10 and 14 days after VEGF-implantation. Panel on left side shows immunoblot and on right side shows quantification of western blotting data collected from corneal tissues of day-10 and day-14. A significant decrease in expression of CD31 on day-10 (62%, p<0.05) and day-14 (66%, p<0.05) was detected in decorin-delivered corneas compared to control corneas suggesting that AAV5-mediated decorin gene therapy is efficient in decreasing CNV. β-actin was used to confirm equal loading of protein in each well and normalization of the data. dcn = decorin.

The efficacy of AAV5-dcn gene therapy was also analyzed by performing western blotting of CD31, a pan-endothelial marker found in blood vessels, platelets, granulocytes, and monocytes that are involved in the formation of new blood vessels. Figure 6 shows results of CD31 western blotting performed with protein lysates prepared from control and decorin-delivered rabbit corneas collected 10 or 14 days after VEGF implantation. As evident from Fig 6, AAV-mediated decorin delivery in rabbit corneas reduced CD31 expression 62±3% (p<0.05) on day-10 and 67±6% (p<0.05) on day14 suggesting that maximal inhibition was attained by day-10 and then continued until the longest tested time point of day-14. The detection of similar intensity β-actin bands confirmed equal loading of protein samples.

### Decorin Gene Therapy Modulates Expression of Pro- and Anti-angiogenic Factors

**Figure 7:**
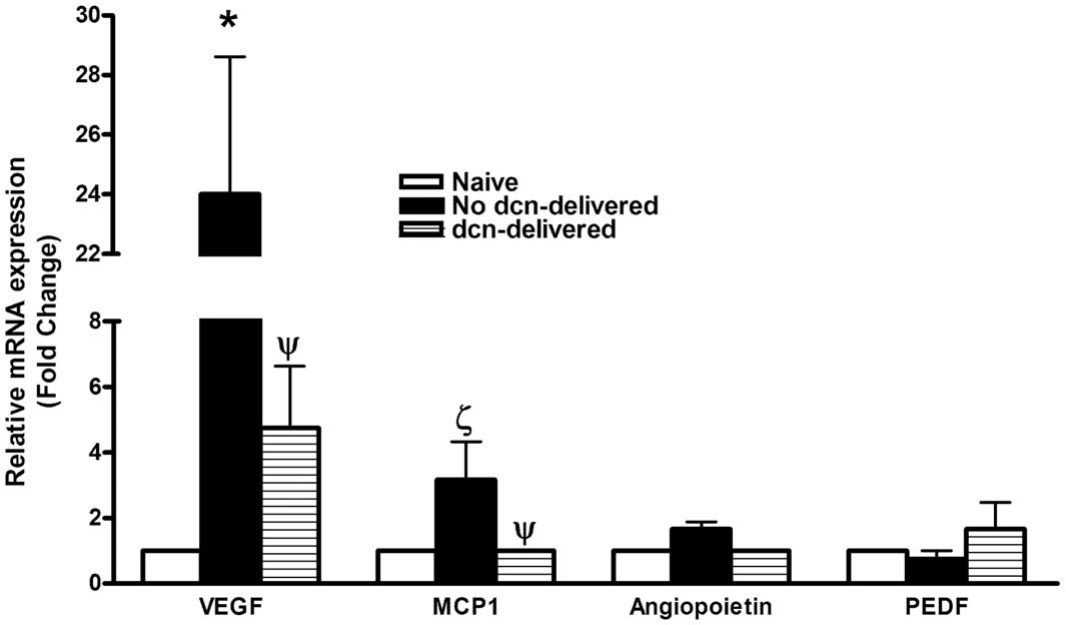
Quantification of mRNA expression of pro- and anti-angiogenic genes by real-time PCR. Real-time PCR was carried out in naive, control (no decorin-delivered) and decorin-delivered rabbit corneal tissues collected 14 days after VEGF implantation. Decorin gene therapy delivered with AAV5 showed marked decrease in the mRNA expression of VEGF and MCP-1 and less pronounced alteration in angiopoietin and PEDF genes. Our data suggests anti-angiogenic effects of decorin gene therapy are mediated by the down-regulation of pro-angiogeneic (VEGF, MCP1 and angiopoietin) and up-regulation of anti-angiogenic (PEDF) genes. * = p<0.001 compared to naive; ζ = p<0.05 compared to naive; ψ = p<0.05 compared to no decorin (dcn)-delivered.

To investigate the possible molecular mechanism of decorin gene therapy for inhibiting CNV, the expression of pro-angiogenic factors such as VEGF, MCP1, angiopoietin and anti-angiogenic factors such as PEDF was analyzed in naive, control (no-decorin) and decorin-delivered rabbit corneas collected 14 days after VEGF-implantation. Figure 7 shows the relative change in the expression of VEGF, MCP1, angiopoietin, and PEDF genes in the cDNA sample of the naive, control (no-decorin) and decorin-delivered rabbit corneas. All cDNA samples with VEGF-induced CNV showed statistically significant increase in VEGF and MCP1 and a less pronounced increase in angiopoietin as compared to cDNA samples obtained from naive corneas. The decorin-delivered corneas showed a significant decrease in VEGF expression (5 fold, p<0.05) and MCP1 (3 fold, p<0.05). Contrary to this, decorin-delivered cornea showed lower angiopoietin and mildly increased PEDF expression, however, data was not statistically significant (Fig. 7).

### Slit-lamp and Optical Coherence Tomography Biomicroscopy and Transmission Electron Microscopy Investigations

**Figure 8:**
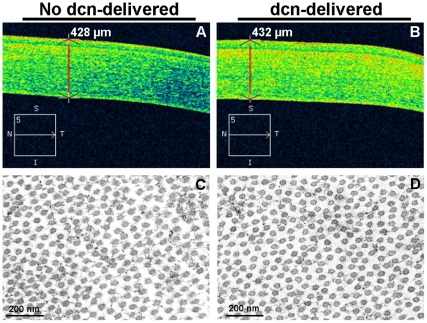
Representative optical coherence tomography (A and B) and transmission electron microscopy (C and D) images of rabbits receiving AAV5-gfp or AAV5-dcn. Optical coherence tomography was performed in live rabbits on day 10 after the AAV5-gfp or AAV5-dcn application and transmission electron microscopy was performed in corneas collected 14 days after AAV5-gfp or AAV5-dcn application. The optical scans of no decorin-delivered (A) and decorin-delivered (B) cornea suggest that tissue-selective targeted delivery of decorin gene with AAV5 in the rabbit stroma does not induce changes in corneal structure, thickness or shape. The TEM did not detect significant differences in collagen fibril diameter and arrangement in the naive (C) and decorin-delivered rabbit corneas (D) indicating that localized therapeutic levels of decorin delivery in the cornea does not appear compromise corneal health. dcn = decorin.

Visual and slit-lamp clinical examination performed in the eyes of live rabbits did not detect any abnormality in non decorin-delivered or decorin-delivered corneas with AAV5 at all tested time points. No haziness, swelling, redness or infection was detected in visual clinical eye examination except in the region where VEGF pellet was implanted. As expected, several blood vessels sprouting from the limbus towards the VEGF pellet were observed in clinical examination. Optical coherence tomography did not detect alteration in corneal structure, thickness or shape of the stroma in decorin-delivered and no decorin-delivered rabbit corneas (Fig 8A and 8B). Furthermore, TEM analysis of naive and decorin-delivered rabbit corneas did not detect any significant differences between the collagen fibril diameter and arrangement in the stroma (Fig 8C and 8D). Slit-lamp, optical coherence tomography and TEM data allowed us to infer that targeted decorin gene transfer into stroma does not affect optical and structural properties of the cornea, and consequently visual function.

## Discussion

Corneal neovascularization is a serious pathological condition often requiring aggressive and prolonged treatment for its effective management. Gene therapy offers a unique opportunity for treating CNV by providing long-term expression of therapeutic genes in the cornea. In this study, we demonstrated significant inhibition of VEGF-induced CNV with decorin gene therapy delivered in rabbit eyes *in vivo* via AAV vector. The vector was administered to the cornea with a custom vector-delivery technique optimized recently in our laboratory [29]. Previously, we demonstrated long-term transgene expression in the cornea with AAV [14]. Recently, we examined the efficacy of multiple AAV serotypes (2, 5, 7, 8 and 9) for corneal gene therapy, and found AAV serotypes 5, 8 and 9 efficient for transducing rabbit and rodent corneas [13]–[18]. In this study, AAV serotype 5 was utilized because this vector was used to optimize targeted delivery of therapeutic genes in the stroma of normal and damaged rabbit corneas [14]. Earlier studies performed with AAV2-mediated anti-angiogenic gene therapy to treat CNV showed 30–50% reduction in CNV [10], [20]. A more robust inhibition of CNV (up to 67%) observed in this study with AAV5 is most likely due to the superior transduction efficiency of the AAV5 serotype. The detection of no apparent side effects in rabbit eye supports our hypothesis that targeted introduction of therapeutic genes in the cornea is an effective approach to treat corneal diseases with minimal side effects.

Decorin is a chondroitin/dermatan sulfate-containing small leucine rich proteoglycan that modulates several essential biological processes in the corneal tissue including collagen fibrillogenesis [21], [22]. Recent studies have implicated the role of decorin in modulation of angiogenesis with decorin over-expression via retroviral transduction showing considerable inhibition of vascular endothelial cell migration [24]. Purified decorin obtained from cartilage and skin tissue has been shown to curtail vascular endothelial cell migration as well as tube formation in an *in vitro* model [23]. In addition to *in vitro* studies, decorin has also been reported to inhibit angiogenesis in tumor xenograft *in vivo* in nude mice [26]. These studies support the results of this present study, which demonstrate significant inhibition of VEGF-induced corneal angiogenesis by decorin gene transfer *in vivo* in a rabbit experimental model of CNV. To the best of our knowledge, this is the first study to demonstrate the potential use of decorin gene therapy for treating corneal angiogenesis *in vivo*. The VEGF-induced corneal micropocket assay used in this study is most reliable and appropriate model for identifying potent anti-angiogenic agents and developing newer strategies for potential therapies [1], [2], [29]–[31]. This *in vivo* model is frequently used in angiogenesis research because of low inflammatory response and minimal confounding effects of wound healing, and allows better understanding of molecular mechanism associated with corneal angiogenesis. VEGF is a known initiator and promoter of angiogenesis, and its implantation in the stroma has been shown to induce robust neovascularization in the cornea by stimulating new blood vessels growth towards the transparent cornea from limbal region [1], [2], [30]. However, it can be argued that in VEGF-induced model angiogenesis is not associated with trauma, injury or infection, which are known to trigger CNV in human patients. In order to fully define clinical translational potential of AAV-decorin gene therapy for treating corneal neovascularization in patients additional *in vivo* studies employing inflammation-based animal model such as alkali burn, chemical cauterization etc are warranted.

Decorin has been shown to interact with a wide variety of bioactive molecules including VEGF [21], [25], [26], [32]. Studies investigating the molecular mechanism of decorin have shown that decorin inhibits angiogenesis by suppressing VEGF or FGF expression [25], [26]. Angiogenesis is a complex process involving interplay between a wide variety of pro- and anti-angiogenic factors [1], [2]. In the present study, the relative expression of well-known pro- and anti-angiogenic genes such as VEGF, MCP1, angiopoietin and PEDF was analyzed to understand the molecular mechanism of decorin-mediated inhibition of CNV. A marked decrease in the expression of VEGF suggests that anti-angiogenic effects of decorin in the rabbit eye are likely due to the attenuation of VEGF. In the cornea, as well as other tissues, it is reported that VEGF stimulates VEGF mostly through paracrine mechanism by recruiting macrophages or increasing expression of other proangiogenic factors that, in turn, enhance VEGF expression [33], [34]. Our study detecting significantly high level of VEGF in VEGF-implanted corneas is in agreement with these literature findings. Furthermore, observed decrease in VEGF mRNA in our study may possibly due to direct inhibition of VEGF gene expression by decorin as reported earlier for non-ocular tissues [26]. It is also possible that decorin may be decreasing VEGF indirectly by modulating other cytokines as decorin is a known ligand for several growth factors and cytokines [22]. This remains to be tested. A small change observed in the MCP1, angiopoietin and PEDF gene expression indicate that they play a less prominent role in decorin-mediated CNV gene therapy in the cornea. More studies to unveil the role of decorin on pro- or anti-angiogenic factors are required to understand the molecular mechanism associated with decorin gene therapy in the cornea. Mimura et al [35] reported that metalloproteinases regulate FGF-induced CNV by causing breakdown of corneal decorin. Therefore, one of the mechanisms by which decorin gene therapy may have prevented angiogenesis is by saturating these metalloproteinases and thus protect endogenous corneal decorin from degradation. Our future studies will address such issues.

Optical coherence tomography imaging is frequently used in ophthalmology clinic to diagnose and image anomalies in eye tissues [36]. The impact of AAV-decorin gene therapy on the cornea and neighboring ocular tissues were recorded and suggested that AAV-mediated decorin gene therapy does not compromise corneal or other ocular tissues. A decorin knockout mice study exhibited that targeted disruption of decorin may compromise collagen fibril morphology and skin fragility [37], [38]. Thus, there exists a theoretical possibility that decorin gene therapy could affect collage organization in the cornea. To address this legitimate concern, we performed transmission electron microscopy studies, and detected no significant differences in collagen fibril diameter and/or arrangement in decorin-delivered rabbit corneas collected after 2 weeks (Fig 8) suggesting that therapeutic levels of delivered decorin does not alter corneal collagens at least for short-term. However, long-term (a year or longer) AAV-decorin toxicity studies are warranted.

In summary, this study demonstrates for the first time significant inhibition of corneal neovascularization in the rabbit cornea with no short-term side effects by AAV5-mediated tissue targeted decorin gene therapy. Furthermore, the results of this study support our hypothesis that tissue-selective delivery of therapeutic genes in the stroma with defined gene therapy approaches may effectively decrease corneal disease severity without insignificant side effects.

## Materials and Methods

### Animals and Micro-pocket Corneal Neovascularization Assay

Twenty-four female New Zealand White rabbits (Myrtle Laboratories Inc., Thompson's Station, TN) weighing 2.5–3.0 kg were used in this study. The Institutional Animal Care and Use Committee of the University of Missouri-Columbia and Harry S. Truman Memorial Veterans' Hospital, Columbia approved the study. Animals were treated in adherence to the ARVO Statement for the Use of Animals in Ophthalmic and Vision Research. Corneal angiogenesis in rabbit eyes was induced by corneal micro-pocket assay [29]. Rabbits were anesthetized by intramuscular injection of ketamine hydrochloride (50 mg/kg) and xylazine hydrochloride (10 mg/kg), and 3–4 drops of 0.5% proparacaine hydrochloride solution (Alcon, Ft. Worth, TX) was topically applied to the eye prior to micro-pocket surgery. Only one eye of each animal was used for the surgical procedure. The contralateral eye served as naive control. A wire speculum was positioned in the eye, and a sucralfate-hydron pellet containing 650 ng of VEGF (PeproTech, Rocky Hill, NJ) was implanted into the cornea after making a micro-pocket in the stroma using standard surgical tools. Triple antibiotic ointment (Alcon) was applied to the surface of the cornea after pellet implantation to prevent infection. The ingrowth of blood vessels in the cornea towards VEGF implant started from day 2, peaked around day 10, and continued to grow progressively up to 15 days before regressing.

### AAV5 Vector Expressing Decorin

Decorin gene was amplified with PCR from the cDNA of primary rabbit corneal fibroblasts using forward (5′GAT CGC GGC CGC AAT CAT GAC GGC AAC TCT CAT C3′) and reverse (5′GTC AGC GGC CGC GAG TTA CTT GTA GTT TCC GAG C3′) primers. The resulting template was cloned into the Not1 site of AAV2 plasmid pTRUF11 containing a hybrid promoter (cytomegalovirus enhancer and chicken β-actin) and simian virus 40 polyadenylation site. The resultant decorin cloned plasmid was packaged into AAV5. In brief, decorin AAV5 vector was produced by the 2-plasmid, co-transfection method [39]. One Cell Stack (Corning Inc., Corning, NY) with approximately 1×10^9^ HEK 293 cells was cultured in Dulbecco's Modified Eagle's Medium (Hyclone Laboratories Inc. Logan UT), supplemented with 5% fetal bovine serum and antibiotics. A CaPO_4_ transfection precipitation was set up by mixing a 1∶1 molar ratio of decorin AAV2 plasmid DNA and AAV5 rep–cap helper plasmid DNA. This precipitate was applied to the cell monolayer and the transfection was allowed to incubate at 37°C, 7% CO_2_ for 60 h. The cells were then harvested and lysed by freeze/thaw cycles and subjected to discontinuous iodixanol gradients centrifugation at 350,000 g for 1 h. This iodixanol fraction was further purified and concentrated by column chromatography on a 5-ml HiTrap Q Sepharose column using a Pharmacia AKTA FPLC system (Amersham Biosciences, Piscataway, NJ). The vector was eluted from the column using 215 mM NaCl, pH 8.0, and the rAAV peak collected. AAV5 decorin (AAV5-dcn) vector-containing fraction was then concentrated and buffer exchanged in Alcon BSS with 0.014% Tween 20, using a Biomax 100 K concentrator (Millipore, Billerica, MA). Vector was titered for DNAse-resistant vector genomes by real-time PCR relative to a standard.

### AAV5 Transduction to Rabbit Cornea

The rabbits were divided into two groups. 100 µl titer (5×10^12^ vg/ml) of AAV5 naked vector or green fluorescent protein gene expressing (AAV5-gfp) vector (n = 12) or AAV5 vector expressing decorin gene (n = 12) was topically applied to the stroma for two minutes one day after VEGF pellet implantation using a custom-delivery technique [32]. Briefly, the corneal epithelium of the rabbit corneas was removed by gentle moving of #64 Beaver blade (Becton–Dickinson, Franklin Lakes, NJ) at 45° angle over the corneal surface under an operating microscope while the animal was under general and local anesthesia. After removing corneal epithelium, eyes were washed with balanced salt solution (Alcon) and wiped with a merocel sponge. A Conair hair dryer of 234 watts (Model 1875; Conair, Stamford, CT) was used for dehydrating the corneas for three cycles of 10 s with 5 s interval after each round. The temperature and air-flow of warm air were 41°C and 6.8 m/s, respectively, according to digital Velocicheck anemometer (Model 8330; TSI Inc., Shoreview, MN). The corneal dehydrating devise was operated from a distance of 8 inches and approximately 45° angle to the eye. Immediately after drying, vector was topically applied using a polystyrene cloning cylinder having 7 mm diameter. The AAV5-gfp or AAV5 naked vector served as a control group to the AAV5-dcn treated group. The contralateral eyes served as a naive control.

### Clinical, Slit-lamp and Optical Coherence Tomography Examinations

The health of the cornea in the eyes of live rabbits was examined by visual clinical and slit-lamp microscopic (BX 900 Slit Lamp, Haag-Streit-USA, Mason, OH) examinations before and after the VEGF pellet implantation or AAV application by two ophthalmologists and a researcher, independently in a blinded manner while animals were under general anesthesia. Thereafter, corneal health was monitored every 3^rd^ day with a hand-held slit-lamp microscope (SL-15, Kowa Optimed Inc., Torrance, CA). Photographs of the cornea were taken with a digital camera attached to the BX 900 slit-lamp microscope. To investigate the corneal structure, corneal thickness and shape of the stroma, optical coherence tomography was performed using Cirrus 3000 high-definition instrument (Carl Zeiss Meditec, Dublin, CA) in control and decorin-delivered rabbits under general anesthesia. The scans with the best signal strength were selected, and imaging data was analyzed with Cirrus optical coherence tomography system software (version 3.0; Carl Zeiss Meditec, Dublin, CA).

### Quantification of Corneal Neovascularization with Stereomicroscopy

The level of neovascularization in the corneas of all 20 rabbits was monitored and quantified with a micrometer-calibrated stereomicroscope (Leica, Wetzlar, Germany) equipped with a digital camera (SpotCam RT KE, Diagnostic Instruments Inc., Sterling Heights, MI). Rabbit eyes were imaged at 5, 10, and 14 days after pellet implantation for determining the efficacy of decorin therapy to inhibit CNV. Adobe Photoshop CS2 (Adobe systems, San Jose, CA) and National Institutes of Health Image J 1.38X (NIH, Bethesda, MD) software were utilized to quantify CNV in the rabbit eye.

### Tissue Collection

Rabbits were humanely euthanized with pentobarbitone (150 mg/kg) overdose under general anesthesia on day 10 (n = 4) or 14 (n = 20) after VEGF pellet implantation. Corneas were removed with forceps and sharp Westcott scissors, embedded in liquid optimal cutting temperature (OCT) compound (Sakura FineTek, Torrance, CA) within a 24 mm×24 mm×5 mm mold (Fisher, Pittsburgh, PA) and snap frozen. Frozen tissue blocks were maintained at −80°C. Tissue sections were cut 7 µm thick with a cryostat (HM 525 M, Microm GmbH, Walldorf, Germany). Sections were placed on 25 mm×75 mm×1 mm microscope Superfrost Plus slides (Fisher), and maintained frozen at −80°C until staining. The excised rabbit corneal tissues were immediately dropped in liquid nitrogen and used for RNA or protein extraction or fixed for TEM analysis. The distribution of 20 rabbits for day-14 was: n = 10 for AAV-gfp, n = 10 for AAV-dcn (6 corneas for immunohistochemistry, 2 corneas for immunoblotting, and one half of 2 corneas for RT-PCR and other half for TEM), and day-10 four rabbit corneas (2 AAV-gfp and 2 AAV-dcn treated) were used for immunoblotting.

### Immunohistochemistry and H&E staining

Corneal tissues were stained for hematoxylin and eosin (H & E), CD31 and collagen type IV to detect CNV area, amount and density. Blood vessel formation was also confirmed with tomato lectin immunostaining. The tissue sections were washed with 1 x HEPES buffer for 15 min, blocked with 5% bovine serum albumin for 30 min, and incubated with 1∶50 dilution of goat polyclonal antibody for collagen type IV (cat # sc-9302; Santa Cruz Biotechnology Inc., Santa Cruz, CA) or CD31 (cat # sc-1506; Santa Cruz) for 90 min followed by donkey anti-goat Alexa 594 secondary antibody (cat # A-11058; Invitrogen, Carlsbad, CA) at 1∶500 dilution for 60 min. Lectin staining entailed the incubation of corneal sections with 20 µg/ml Texas red-conjugated tomato lectin (cat # TL-1176; Vector laboratories, Burlingame, CA) for 90 min. Tissue sections were washed in HEPES buffer and mounted using Vectashield medium containing 4′-6-diamidino-2-phenylindole (DAPI; Vector laboratories). The stained sections were viewed and photographed with a Leica fluorescent microscope (Leica DM 4000B; Leica) equipped with a digital camera (SpotCam RT KE).

### Immunoblotting

The protein lysates were prepared by homogenizing corneas in protein lysis buffer (5 mM EDTA, 150 mM NaCl, 0.002 M phenylmethylsulfonyl fluoride, 50 mM Tris-HCl, pH 7.5) containing 1 µL protease inhibitor cocktail (Roche Applied Sciences, Indianapolis, IN). The sample was homogenized and incubated at 37°C for 30 minutes after adding 1 µL Nonidet P-40, 1 µL Tween 20, and 1 µL 20% sodium dodecyl sulphate, and centrifuged at 16000 g for 15 minutes at 4°C. The same amount of protein of each sample was suspended in Laemmli denaturing sample buffer, vortexed and heated for 10 min at 70°C. The proteins were resolved on 4–20% SDS-PAGE gel and transferred onto 0.45 µm pore size PVDF membrane (Invitrogen, San Diego, CA). The membrane was incubated with decorin (cat # sc-73896; Santa Cruz) or CD31 (cat # sc-1506; Santa Cruz) or β-actin (cat # sc-69879; Santa Cruz) or GAPDH (cat# sc-48166; Santa Cruz) primary antibody followed by alkaline phosphatase-conjugated anti-goat or anti-mouse secondary antibody (Santa Cruz). The bands were visualized by NBT/BCIP. The gel data was analyzed using National Institutes of Health Image J 1.38X (NIH, Bethesda, MD) software.

### RNA Extraction, cDNA Synthesis, and Real Time PCR

Corneal tissues were homogenized in RLT buffer using TissueLyser (Qiagen Inc., Valencia, CA) for 2 min at 20 Hz. Total RNA was extracted using RNeasy kit (Qiagen Inc., Valencia, CA), and reverse-transcribed to cDNA following vendor's instructions (Promega, Madison, WI). Briefly, tissues were lysed in 350 µl RLT buffer (Qiagen Inc., Valencia, CA) containing β-mercaptoethanol followed by 590 µl RNase free water containing proteinase K solution. The solution was incubated at 55°C for 10 min and centrifuged at 10,000 g for 5 min. Half the volume of ethanol was added to supernatant and resulting solution was applied onto RNeasy column. The RNA was eluted with 30 µl of RNAse free water and reverse transcribed to cDNA using a commercial kit following vendor's instructions by carrying reverse transcription reaction at 42°C for 30 min followed by enzyme inactivation with heat at 90°C for 2 min (Promega). Semi-quantitative PCR reactions were performed to determine VEGF, macrophage chemoattractant protein (MCP1), pigmented epithelium-derived factor (PEDF), and angiopoietin levels. A 20 µl reaction mixture containing 2 µl cDNA, 2 µl forward (200 nM) and 2 µl reverse (200 nM) primer, 3.125 mM of deoxynucleotide triphosphates (dNTPs) and Taq polymerase was run one cycle at 95°C for 3 min, then 40 cycles of 95°C 30 s, followed by 55°C 30 s and 55°C for 60 s, using a thermocycler (Bio-Rad Laboratories, Hercules, CA). The VEGF forward and reverse primer sequences were 5′-ACC CAT GGC AGA AGA AGG AGA CAA-3′ and 5′-ACT CCA GGC TTT CAT CAT TGC AGC-3′, the MCP1 forward and reverse primer sequences were 5′-TCG CTC AGC CAG ATG CAA TCA ATG-3′ and 5′-TGG AAT CCT GAA CCC ACT TCT GCT-3′, the PEDF forward and reverse primer sequences were 5′-TGA TGT CGG ACC CTA AGG CTG TTT-3′ and 5′-ATG AAT GAA CTC GGA GGT GAG GCT-3′, the angiopoietin forward and reverse primer sequences were 5′-TTT GCT TTC CTC GCT GCC ATT CTG-3′ and 5′-CAC ATT GCC CAT GTT GAA TCC GGT-3′ whereas the β-actin (a house keeping gene) gene's forward and reverse primer sequences were 5′-CGG CTA CAG CTT CAC CAC CA-3′ and 5′-CGG GCA GCT CGT AGC TCT TC-3′. Each PCR experiment was repeated at least three times.

### Transmission Electron Microscopy (TEM)

Rabbit corneas were fixed in TEM fixative and processed at the University of Missouri Electron Microscopy Core. The 85 nm ultrathin corneal sections were prepared using Leica Ultracut UCT ultramicrotome and transferred onto a 200 mesh copper grid for post-staining with uranyl acetate and Sato's triple lead stain. The samples were then imaged using JEOL 1400 transmission electron microscope (Tokyo, Japan).

### Statistical Analysis

The CNV morphometry and quantitative PCR results are expressed as mean ± standard error of the mean (SEM). Statistical analysis of angiogenesis and PCR data was performed using two-way analysis of variance (ANOVA) followed by Bonferroni multiple comparisons test. A value of p<0.05 was considered statistically significant.
